# The Rapid Emergence of Hypervirulent *Klebsiella* Species and *Burkholderia pseudomallei* as Major Health Threats in Southeast Asia: The Urgent Need for Recognition as Neglected Tropical Diseases

**DOI:** 10.3390/tropicalmed9040080

**Published:** 2024-04-08

**Authors:** Matthew J. W. Kain, Nicola L. Reece, Christopher M. Parry, Giri Shan Rajahram, David L. Paterson, Stephen D. Woolley

**Affiliations:** 1 Institute of Naval Medicine, Alverstoke, Hampshire PO12 2DL, UK; 2 Headquarters Army Medical Services, Camberley GU15 4NP, UK; 3 Department of Clinical Sciences, Liverpool School of Tropical Medicine, Liverpool L3 5QA, UK; christopher.parry@lstmed.ac.uk; 4 Centre for Tropical Medicine and Global Health, Nuffield Department of Clinical Medicine, University of Oxford, Oxford OX1 2JD, UK; 5 Department of Medicine, Queen Elizabeth II Hospital, Kota Kinabalu 88300, Malaysia; 6 Infectious Diseases Society, Kota Kinabalu Sabah-Menzies School of Health Research, Clinical Research Unit, Kota Kinabalu 88994, Malaysia; 7 ADVANCE-ID Network, Saw Swee Hock School of Public Health, National University of Singapore, Singapore 119077, Singapore; 8 Infectious Diseases Translational Research Programme, Yong Loo Lin School of Medicine, National University of Singapore, Singapore 119077, Singapore; 9 Tropical and Infectious Diseases Unit, Liverpool University Hospitals Foundation NHS Trust, Liverpool L7 8YE, UK

**Keywords:** NTDs, neglected tropical disease, melioidosis, *Klebsiella pneumoniae*, *Burkholderia pseudomallei*, hypervirulent

## Abstract

The World Health Organization (WHO)’s list of neglected tropical diseases (NTDs) highlights conditions that are responsible for devastating health, social and economic consequences, and yet, they are overlooked and poorly resourced. The NTD list does not include conditions caused by Gram-negative bacilli (GNB). Infections due to GNB cause significant morbidity and mortality and are prevalent worldwide. Southeast Asia is a WHO region of low- and middle-income countries carrying the largest burden of NTDs. Two significant health threats in Southeast Asia are *Burkholderia pseudomallei* (causing melioidosis) and hypervirulent *Klebsiella pneumoniae* (HvKp). Both diseases have high mortality and increasing prevalence, yet both suffer from a lack of awareness, significant under-resourcing, incomplete epidemiological data, limited diagnostics, and a lack of evidence-based treatment. Emerging evidence shows that both melioidosis and HvKp are spreading globally, including in high-income countries, highlighting the potential future global threat they pose. In this article, we review both conditions, identifying current trends and challenges in Southeast Asia and areas for future research. We also argue that melioidosis and HvKp merit inclusion as NTDs, and that mandatory global surveillance and reporting systems should be established, and we make an urgent call for research to better understand, detect, and treat these neglected diseases.

## 1. Introduction

The global effort to control infectious diseases, especially in Southeast Asia, has focused on reducing tuberculosis, HIV, and malaria. In 2020, the World Health Organization (WHO) produced a roadmap identifying 20 neglected tropical diseases (NTDs), aiming to eradicate these diseases globally by 2030 [1]. NTDs are defined by the WHO as a diverse range of conditions caused by a variety of pathogens (viruses, bacteria, parasites, fungi, and toxins) and associated with devastating health, social, and economic consequences. The WHO list currently sits at 21 conditions that are recognised as NTDs; however, it is felt by many that this list is too small, and other conditions should be considered on this list, including melioidosis [2]. Southeast Asia has been identified as the WHO region with the largest NTD burden, with 857 million individuals requiring interventions. Yet, the current WHO list of NTDs does not include any Gram-negative bacilli (GNB). In particular, melioidosis and hypervirulent *Klebsiella pneumoniae* cause significant morbidity and mortality across Southeast Asia and we argue that they meet the criteria to be listed as NTDs. The data on the incidence of melioidosis are scant; however, there are estimated to be 165,000 cases annually, with nearly 90,000 deaths [3]. Although these data are likely to represent a significant underestimate, it is known that Southeast Asia is the region most significantly affected by melioidosis. Another GNB which has significant health implications in the region is hypervirulent *Klebsiella pneumoniae* (HvKp). First emerging in Taiwan in the 1980s, HvKp has rapidly spread throughout Asia, including Southeast Asia. The true burden of disease, especially in low-resource settings, remains unknown. HvKp can result in mortality ranging from 10–40% in community-acquired infections [4] and up to 80% in hospital-acquired infections [5]. Classic HvKp is usually fully susceptible to most used antimicrobials, although multi-drug resistant strains are being increasingly detected. *Burkholderia pseudomallei* (the causative agent of melioidosis) has a unique antibiogram but acquired resistance is rare [6].

With both diseases, there is a paucity of evidence, especially surveillance data, supporting diagnostics and treatment. There are unanswered questions regarding both diseases, such as the benefit of monotherapy versus dual therapy and the role of adjuvant therapies in addition to antimicrobials. In this article, we discuss both diseases, highlight the case for both to be included in the WHO NTD list, and propose a strategy to tackle these neglected pathogens.

## 2. Melioidosis

### 2.1. Introduction

*Burkholderia pseudomallei* (*B. pseudomallei*) is a GNB that resides in moist soil and ground water, mostly in tropical climates. It is the causative pathogen of melioidosis. Human infection is usually acquired through the inoculation of the bacilli through the skin; inhalation and ingestion are also routes of exposure [7]. Although the bacterium has been recognised for over 100 years, its epidemiology is not well understood, and questions remain about the optimal way to diagnose and treat this condition.

### 2.2. Epidemiology

*B. pseudomallei* is found throughout the tropical and subtropical regions. Southeast Asia has an estimated 44% of the world’s global disease burden [3]. The prevalence of *B. pseudomallei* is difficult to calculate due to the lack of mandatory reporting systems in many countries with high burdens of disease. Melioidosis is a notifiable condition in Australia, Singapore, and Thailand; it is an administratively notifiable disease in Sabah, Malaysia; however, other countries do not have compulsory notification systems in place [8]. It is also poorly recognised by many clinicians and laboratory staff, contributing to significant under-reporting. A modelling paper estimated that *B. pseudomallei* would be responsible for 165,000 cases and 89,000 deaths globally in 2015. This study suggested that the highest rates of melioidosis would be found in areas with environmental suitability: high rainfall, warmer temperatures, high soil salinity, and certain types of soil, such as those rich in clay. Low country incomes and areas with a high prevalence of diabetes mellitus were also associated with higher rates of melioidosis [3].

A more recent review paper estimated the overall prevalence of *B. pseudomallei* in Southeast Asia to be between 0.02% and 74.4% [9]. There is variation in the burden of disease between different geographic locations and patient groups; for example, in rural northeast Thailand, a study found an annual incidence of bacteraemic melioidosis to be 14–17 per 100,000 [10], whereas a study of children with suppurative parotitis in Cambodia found 74% had culture-confirmed *B. pseudomallei* [11]. A further study looking at the causes of sepsis in teenagers and adults from Yangon, Myanmar found *B. pseudomallei* only responsible for 1.1% of cases [12]. It is not clear if this heterogeneity of epidemiology is due to true variations in disease burden or due to difficulties in detecting the organism and confirming the diagnosis. This highlights the need for robust surveillance systems. Although melioidosis was first reported over 100 years ago, the true prevalence has been historically difficult to determine prior to the development of local internal laboratory systems with the ability to perform bacterial culture at scale.

Melioidosis mainly affects rural agricultural workers, especially those who work in waterlogged areas such as rice paddies [3]. Cases peak during monsoon seasons or after extreme weather events when *B. pseudomallei* is forced to the surface of the soil, increasing the risk of infection [7,13].

Basic prevention measures, such as clean drinking water, waterproof knee-high boots, and improved glycaemic control, are likely to significantly reduce cases [14]. However, many of these precautions are not practised by at-risk groups [15]. Widespread chlorination or the UV light treatment of drinking water may help reduce infections caused by ingestion but has little impact on the majority of cases who are infected via skin inoculation, especially feet, and is unlikely to be feasible in resource-limited settings [13]. Furthermore, there is a lack of awareness of melioidosis in the at-risk community. A study in northwest Thailand amongst diabetic rural agricultural workers found that 97% had no awareness of melioidosis, although the reasons for this are unclear [15]. As the disease is not spread person-to-person, large-scale epidemics do not occur, meaning case numbers in discrete locations may stay relatively low. This is further compounded by the challenges of diagnosis, meaning the proportion of the at-risk population that ever receives a formal diagnosis of melioidosis is likely to be very small. In addition, large-scale public health information campaigns may be harder to establish due to the heterogenicity of acquisition and clinical presentation of the disease. Further research is required to fully understand why there is such low awareness of the condition amongst at-risk populations, which could also help to develop successful future public health interventions.

### 2.3. Clinical Syndrome

Melioidosis is known as the great mimic and can present with a range of symptoms. Many immunocompetent individuals will have asymptomatic disease and successfully clear the infection. There is a strong association with diabetes, with melioidosis ten times more likely to occur in people with type 2 diabetes than non-diabetics. This is due to a combination of dysregulated immune responses including poor IL-12 production, which is linked to a lack of intracellular glutathione and reduced bacterial cell killing [16]. Reduced macrophage function, decreased production of regulatory CD4+ cells, and reduced CD4+ T cell function also seem to contribute to the excess disease seen in diabetic patients. [7,17]. There is also a weaker association with chronic renal failure and with alcoholism [13]. Fever is the most common symptom associated with melioidosis. A review of cases from Sabah, Malaysia also found that fever was significantly associated with patients being bacteraemic [18]. Localised cutaneous disease, such as rash or abscess, commonly occurs following skin inoculation and accounted for 50% of all cases in a retrospective study in Southern Thailand [19]. In those with severe disease, most patients present with sepsis with or without pneumonia, which is independent of the route of exposure [7,18]. Other common presentations include visceral abscess formation (for example, in the liver, spleen, urinary tract, and prostate) [20,21]. Due to the wide variety of presentations, it is very challenging to diagnose melioidosis clinically [7].

Recurrence is estimated to occur in 5–28% of patients. This is most commonly seen in the form of relapse in patients who have not completely cleared their disease. Research suggests that this is partly due to bacterial adaptative changes leading to increased antibiotic resistance, although the bacterial mechanisms contributing to persistent infections are not yet fully understood [22]. Reported mortality varies but is estimated to be 30–50%. With improvements in recognition and management in Northern Australia, this has fallen to 6% [23,24].

### 2.4. Diagnosis

Microbiological diagnosis is highly challenging. Although *B. pseudomallei* grows easily on horse blood or chocolate agar, it may be overgrown by other organisms. Additionally, it can look similar to Pseudomonas species, particularly *Pseudomonas stutzeri*, and, therefore, it may be dismissed as an environmental contaminant [25]. This is compounded by a lack of awareness of the bacterium amongst laboratory staff and by the fact that only 50–75% of patients are bacteraemic at the time of presentation [26]. The gold standard of diagnosis is culture from any human sample, grown on selective media. The antibiogram is very characteristic with intrinsic resistance to gentamicin and colistin but susceptibility to co-amoxiclav. This can be combined with the Gram stain and a positive oxidase test to presumptively identify the organism [7].

The European Committee on Antimicrobial Susceptibility Testing (EUCAST) has recently developed a minimum inhibitory concentration (MIC) and zone diameter breakpoints for *B. pseudomallei*; however, these include an “I” category for “susceptible, increased exposure”. This new category in other bacterial organisms has caused global confusion amongst clinicians and requires education to ensure the most suitable drug is used and organisms labelled “I” are not treated as resistant [27]. The Clinical and Laboratory Standards Institute (CLSI) recommends a broth dilution method which has been calibrated for relevant antibiotics [28]. However, many laboratories in *B. pseudomallei* endemic regions do not have the expertise and infrastructure to perform broth dilution testing. Matrix-assisted laser desorption/ionisation time-of-flight mass spectrometry (MADLI- TOF) systems are increasingly used for organism identification, and this may explain the increased detection of *B. pseudomallei* in areas where the bacterium has not previously been identified. Detection via MALDI-TOF generally has high sensitivity and specificity; however, occasionally, *B. pseudomallei* is misidentified, most frequently as *Burkholderia thailandensis*. Additionally, MALDI machines are expensive, require reliable electricity and maintenance, and are unlikely to be available in rural laboratories where most cases occur [29]. Compounding this, appropriate laboratory infrastructure should be available to handle *B. pseudomallei* as it is a hazard group 3 pathogen [30].

Serology is of limited use in melioidosis due to the lack of international standardisation. A variety of assays target differing antigens with variable sensitivities and specificities. The traditional serological assay used is the indirect haemagglutination assay, but it is poorly standardised as it uses rabbit red blood cells sensitised to crude *B. pseudomallei* antigens. Newer serological assays using enzyme-linked immunosorbent assay (ELISA) for the detection of antibodies to the O polysaccharide or haemolysin-coregulated protein (Hcp1) have better sensitivity but are challenging to interpret in endemic areas where there is high background seropositivity [8]. PCR is available to detect *B. pseudomallei*, but its high genetic variation and low levels of colony-forming unit (CFU) in the bloodstream result in low sensitivity, limiting its use. PCR is also expensive and unlikely to be available in lower-resource settings [7,13].

More recently, a lateral flow assay (LFA) detecting *B. pseudomallei* capsular polysaccharide using a monoclonal antibody has been developed (Active Melioidosis Detect (AMD LFA)). The test is easy to carry out and can be performed with minimal training. The result is available within an hour and is cheap at approximately USD 2 per test [31]. In a review of eight studies, 56% of melioidosis patients were positive on one or more AMD tests, increasing positivity was seen in patients with a higher burden of disease, and AMD reached sensitivities of >80% in sputum and pus that were culture positive. Some limitations of the test are that it cannot distinguish between dead and viable bacteria, and there is limited data on its specificity [32].

*B. pseudomallei* commonly causes abscesses which can be present in a variety of organs. Access to cross-sectional imaging to investigate is essential, especially for the liver, spleen, urinary tract, and prostatic foci [33]. Identifying abscesses helps to establish the diagnosis of melioidosis but requires sophisticated radiology techniques such as computed tomography (CT) along with staff trained in how to interpret images [13,20]. Furthermore, radiological imaging is critical to establish the presence and site of infection in order to guide the treatment duration. These imaging platforms are unlikely to be available in resource-limited or remote rural settings.

### 2.5. Treatment

Current treatment regimens are divided into two phases (Table 1): an intensive phase of intravenous therapy, usually with ceftazidime or meropenem for at least 14 days, followed by an eradication phase for a minimum of 3 months (Table 2) [34,35].

Ceftazidime or meropenem are the drugs of choice for the intensive phase of treatment. In Thailand in 2016, a 14-day course of ceftazidime cost USD 60, and meropenem cost USD 1080. Depending on how healthcare is funded, many patients or hospitals in lower-income countries may find this unaffordable, whilst drug availability can also be an issue in remote settings [7]. Evidence is conflicted as to whether meropenem or ceftazidime is better in the intensive phase. Data from Australia suggest meropenem has a survival benefit in patients requiring the intensive care unit (ICU). It is not clear if this is also true for non-ICU patients [36,37]. Studies have looked at reducing the duration of the eradication phase to less than 3 months, but relapse is common, especially in those treated for less than 8 weeks [37]. Recent data suggest that having a longer intensive phase may allow for shorter oral continuation therapy, which could improve compliance, but this has yet to be confirmed in further studies [38]. Up to 40% of patients have adverse effects during the course of therapy. Fortunately, drug-resistant *B. pseudomallei* appears to be rare. Some studies suggested co-trimoxazole resistance was common; however, this is likely to be due to difficulties interpreting disk diffusion rather than true resistance [39]. Sporadic cefiderocol resistance has been reported from parts of Southeast Asia, including Thailand and Malaysia, which may have implications for patients who develop resistance to first-line treatment options [40]. Cefiderocol is not commonly used in Southeast Asia; therefore, the emergence of this resistance pattern is particularly concerning and requires further evaluation.

Source control is also an essential part of treatment if abscesses are present, and this requires surgical infrastructure and expertise [13,23]. There is a paucity of data regarding adjunctive therapy. Observational data suggested that granulocyte colony-stimulating factor (G-CSF) increased survival, but a large randomised controlled trial (RCT) in Thailand did not replicate this finding [41]. Anecdotally, G-CSF is still commonly used in critically unwell patients in high-resource settings although the evidence base for this is lacking. Specifically, multinational RCTs examining the effect of adjunctive therapies in different clinical settings are required [41,42].

### 2.6. Challenges

There are many challenges to combating melioidosis. We do not clearly understand the epidemiology or the reasons behind the emergence of this disease in new countries. The burden of melioidosis is likely to increase over the coming decades partially due to rising rates of diabetes in addition to environmental factors which may also have an impact. It has been shown the seroprevalence of melioidosis increased after a large tsunami in Thailand [43]; therefore, an increasing frequency of severe weather events secondary to climate change may increase the population at risk from *B. pseudomallei*.

Most melioidosis occurs in the world’s rural poor, who have the least access to hospitals and laboratories able to diagnose and treat this condition successfully. The lack of awareness amongst clinical and laboratory staff contributes to poor outcomes. Gold-standard diagnostic methods require sophisticated laboratories with well-trained staff who understand the bacteria, its growth requirements, and classic antibacterial sensitivity patterns [13].

Management requires prompt treatment with an extended period of intravenous antibiotics and, in many cases, advanced critical care facilities that may not be available in resource-limited settings. First-line antibiotics are expensive and may not be accessible to remote hospitals. The eradication stage requires a prolonged course of antibiotics, with the associated problems of adherence and adverse effects, and the risk of relapses. Best-practice treatment regimens, including the duration of antibiotic treatment and the use of adjuncts, have not been clearly established by RCTs.

There is a lack of awareness of the disease amongst at-risk populations, and public health measures such as boiling water and wearing rubber boots whilst outside are not widely practised [7].

*B. pseudomallei* also has the potential to be used as a biological warfare agent, leading to mass casualties which could overrun healthcare facilities, even in well-resourced countries [44]. There are several vaccine candidates, including one targeting a capsular polysaccharide and another based on outer membrane vesicles, which are scheduled to start phase 1 clinical trials. These are unlikely to be clinically available in the near future [32].

## 3. Hypervirulent *Klebsiella pneumoniae*

### 3.1. Introduction

*Klebsiella pneumoniae* is a Gram-negative, encapsulated rod of the Enterobacterales family found in soil, water, and animals. The organism colonises human skin, the nasopharynx, and the gastrointestinal tract, especially in healthcare settings. Classical *K. pneumoniae* (cKp) infection commonly results in pneumonia, urinary tract infections, and bacteraemia, typically in immunocompromised patients in healthcare settings [45]. HvKp was first described in 1986, in a case series of seven healthy patients with primary liver abscess and septic endophthalmitis [46]. Subsequent research has identified HvKp as a separate pathotype from cKp that has unique epidemiology and is more virulent than cKp [47]. HvKp most commonly causes community-acquired infection in healthy individuals and is also associated with diabetes mellitus [48,49,50,51]. It typically causes cryptogenic, rapidly progressive, and metastatic abscess-forming disease, which is often fatal [52]. The remarkable ability of *Klebsiella pneumoniae* to share genetic information has led to the convergence of cKp and HvKp, leading to multi-drug resistant (MDR), hypervirulent strains [53] that pose an unmet, global health threat.

The individual contribution of virulence factors towards the hypervirulence phenotype is complex and reviewed extensively elsewhere [45]. Virulence plasmids are the best-described mechanism of the acquisition of hypervirulence [54,55]. Nevertheless, recent studies have shown that virulence plasmids are not always present in hypervirulent strains [56], with integrative conjugative elements and other mechanisms playing a significant role in the acquisition of virulence factors [53].

Key virulence factors include the development of a bacterial polysaccharide hypermucoid capsule and increased iron acquisition [45]. The bacterial capsule interacts with the external environment and protects against phagocytosis and complement-mediated activity [57]. The capsule serotypes K1 and K2 are most commonly associated with HvKp [4]. HvKp typically has an overproduction of polysaccharide capsule, upregulated by virulence factors such as rmpA/rmpA2 [55,58], providing enhanced protection from neutrophil-mediated killing [59], and commonly resulting in a hypermucoviscous (HMV) phenotype [57,58].

Iron is essential for bacterial metabolism. In extracellular bacterial infection, the host responds by restricting the availability of free iron via hepcidin synthesis [45,60]. To counter this, *Klebsiella pneumoniae* secretes four siderophores, which can efficiently sequester both free and bound iron. The siderophores aerobactin and salmochelin are highly expressed in HvKp compared to cKp [52] and are associated with hypermucoid capsule production [61]. A high total siderophore concentration is also strongly predictive of HvKp [62].

### 3.2. Epidemiology

HvKp is predominantly found in Asia. It was first discovered in Taiwan [46], with populations in Asia having the highest known prevalence rates [51]. Interestingly, in the West, HvKp infections are more common in those of Asian ethnicity [63,64], whilst in Singapore, patients of Chinese ethnicity had higher rates of virulent *Klebsiella pneumoniae* disease than the non-Chinese [65]. The reasons for these associations are unknown. Environmental transmission, with or without genetic susceptibility, is likely responsible. Residents of Southeast Asian countries have particularly high rates of *Klebsiella pneumoniae* colonisation in stool, including capsular serotypes associated with hypervirulence [66]. Hypervirulent stains have also been observed to transmit amongst families, causing disease [67]. A genetic predisposition has been hypothesised, but no convincing evidence exists to support this.

The true, or estimated, burden of HvKp disease in either Southeast Asia or globally is unknown. HvKp cases have been reported throughout Southeast Asia, for example, in Malaysia [68,69], Myanmar [70], Singapore [65,71], and Thailand [72]. A study investigating the prevalence of virulence genes in *Klebsiella pneumoniae* bloodstream infections from India, Nepal, Vietnam, Thailand, Laos, Cambodia, and Hong Kong found virulence genes in 28% of isolates, more than double the rate seen in similar studies focused outside of Southeast Asia [73,74].

Southeast Asia predominantly consists of low- and middle-income countries (LMICs). Resource limitations can hamper their ability to detect and report disease, especially in rural areas. For example, significant data gaps exist in the epidemiology of antimicrobial-resistant Enterobacterales in Southeast Asia [75]. Many HvKp cases likely go undetected or unreported in these countries, in contrast to high-income neighbours such as Taiwan and South Korea. Between 1996 and 2004, an almost 60% increase in the annual incidence of liver abscesses caused by HvKp was observed in Taiwan [76]. In South Korea, the proportion of liver abscesses caused by *K. pneumoniae* rose from 3.3% in the 1970s to 78.2% in the mid-2000s [77]. Singapore, a Southeast Asian high-income country (HIC), has similarly high rates of cryptogenic *K. pneumoniae* liver abscess [78]. Whether the wealth of epidemiological data from HIC Asian Pacific Rim and Southeast Asian countries, compared to LMICs, is due to more robust case detection and reporting, is unknown.

Cases of HvKp are being reported more frequently globally, in wider Asia [71,79,80,81], Western Europe [82,83,84,85,86], Scandinavia [87,88], North America [63,88,89,90], South America [91], and Southern Africa [51,88]. This shows an ability for HvKp to spread into new environments and populations.

### 3.3. Clinical Syndrome

Following colonisation, what triggers HvKp to cross the gastrointestinal mucosal and epithelial barrier to cause infection is unclear. Colonisation with HvKp, compared to cKp, is more likely to result in infection [92]. Serosurveillance studies have shown that Southeast Asian populations have a high prevalence of virulent-serotype *Klebsiella pneumoniae* colonisation in stool [66]. Infection mainly occurs in healthy hosts [93], without evidence of gastrointestinal mucosal disruption. Increasing the *K. pneumoniae* burden within the gastrointestinal system through selection after antibiotic administration increases the risk of infection [94]. Pulmonary disease and hospital-acquired infection are caused by aspiration and the introduction of invasive devices, respectively.

The presentation of HvKp is non-specific but classically presents as bacteraemia and hepatic abscess, often rapidly metastasising to distant sites, with septic shock that is potentially fatal in young, immunocompetent patients [49]. Hepatic abscesses are typically solitary and often associated with hepatic thrombophlebitis, splenic abscesses, and normal biliary anatomy [93,95]. However, presentations are varied. Bacteraemia is common and may be primary (without any obvious cause) or secondary to liver abscess [96]. Patients may also show signs of metastatic spread at presentation, most commonly endophthalmitis, meningitis, and lung abscesses [97]. The mechanism by which HvKp has a predilection for metastatic spread over cKp is unknown [93]. The effects, particularly of endophthalmitis, can be devastating [98]. The benefit of routine ophthalmological assessment in the presence of a liver abscess, or the benefit of routine liver imaging in the presence of endophthalmitis, has not been established and warrants further investigation.

### 3.4. Diagnosis

*Klebsiella pneumoniae* can be readily identified in blood or tissue sampling by standard microbiological techniques. Differentiating HvKp from cKp can be more challenging, especially so in resource-limited, rural areas of Southeast Asia. Bioassays, such as BALB/c mice lethality models [47] and Galleria mellonella lethality models [99], have been used, but are not practical and have varying results [99,100].

A positive string test (i.e., when viscous strings ≥5 mm in length are formed when a wire loop is used to stretch a colony on an agar plate) was traditionally used to identify an HMV phenotype [51]. HMV strains correlated with higher serum resistance and were more prevalent in invasive infection than non-HMV strains [4]. However, the HMV phenotype and string test were neither sensitive nor specific for HvKp [62], and the need for more accurate biomarkers has been identified [101].

Predictive biomarkers for HvKp (peg-344, iroB, icuA, rmpA, rmpA2, and total siderophore production >30 μg/mL) have been shown to differentiate HvKp from cKp accurately [62]. A multiplex PCR was validated to identify certain carbapenem-resistant (CR) HvKp strains [102]. These methods have limitations as the genetic determinants of a hypervirulent phenotype are complex, and biomarkers may not always be expressed [103]. They are also expensive and require significant laboratory infrastructure. In the absence of reliable and accessible diagnostics, the clinical features and epidemiology of HvKp remain important tools to differentiate HvKp from cKp; but, with changing epidemiology, the need for an accurate and practical test to differentiate HvKp is required.

### 3.5. Treatment

The treatment of HvKp is difficult and lacks high-quality evidence in Southeast Asia and globally. In undifferentiated *K. pneumoniae* liver abscess in Singapore, the A-KLASS trial [104] established oral therapy as non-inferior to intravenous therapy. The generalisability of these results to patients with metastatic HvKp in settings of high antimicrobial resistance is unclear. Empiric antibiotic treatment will depend on the site of infection (for antibiotic tissue penetration) and the likelihood of antibiotic resistance [93]. Further, the high frequency of HMV phenotype amongst HvKp strains makes draining viscous abscess contents difficult, necessitating large bore drains. There is a lack of studies assessing the optimum method of source control (antibiotics alone vs. percutaneous drainage vs. surgical drainage) specifically for HvKp [93]. Another therapeutic question that needs answering is the benefit of antibiotic monotherapy vs. dual therapy in complicated infections.

The development of drug resistance in HvKp strains is of particular concern. Historically, HvKp has harboured less antibiotic resistance than cKp [49,105]. MDR-cKp is genetically diverse, with frequent losses and gains of plasmids [106]. In contrast, HvKp may have a restriction on horizontal gene transfer, possibly hampered by the hypercapsule [45], though the acquisition of resistance plasmids by HvKp does occur [107]. As a result, MDR-cKp appears to acquire HvKp virulence factors more easily than HvKp acquires resistance plasmids [101,106]. In addition, cKp may acquire the hypermucoid phenotype independent of plasmid-borne virulence genes such as rmpA, through mutations in chromosomal wzc [108].

The confluence of multi-drug resistance and hypervirulence has led to the development of extended-spectrum β-lactamase-producing HvKp (ESBL-HvKp) [49,109] and hypervirulent carbapenem-resistant *Klebsiella pneumoniae* (Hv-CRKp) strains [49,72,103]. These MDR-HvKp strains have been detected in Southeast Asia [68,72] and are spreading worldwide [49,79,103,109,110,111].

MDR-HvKp is becoming more prevalent in nosocomial infections, affecting more co-morbid patients, and blurring the lines between the traditional clinical and epidemiological differentiation of HvKp from cKp. Carbapenem-resistant cKp strains that have acquired hypervirulence plasmids (Hv-CRKp) are more effective at colonising hospital settings than HvKp strains that have acquired carbapenem resistance (CR-HvKp); Hv-CRKp is becoming the dominant hypervirulent, carbapenem-resistant *K. pneumoniae* in hospital settings [112].

The prevention of the spread of MDR-HvKp is essential. CR-HvKp and Hv-CRKp strains survive better in both community and healthcare settings, due to their extreme drug resistance and virulence [45]. In intensive care units (ICUs), they are 3.7 times more transmissible than *Escherichia coli* [113]. Effective infection prevention and control (IPC) is essential for all drug-resistant Enterobacterales but may also be required for drug-sensitive HvKp to prevent the transfer of virulence factors [93]. In addition, effective surveillance systems are required to successfully identify CR-HvKp strains [114].

### 3.6. Challenges

Since the rapid emergence of HvKp in the 1980s, much research has focused on understanding the mechanisms of virulence. Yet, fundamentally, there remains a lack of understanding of the epidemiology, diagnostics, and effective treatment of HvKp. Research and clinical practice are hampered by the lack of a global definition or accurate methods of the distinction of HvKp from cKp. These diagnostics are urgently required, especially in laboratories with limited resources, such as many in Southeast Asia [115].

As previously discussed, HvKp is present in Southeast Asia [66]. Studies have shown higher rates of cKp and HvKp carriage in stool in LMICs compared to HICs [116,117]. In Southeast Asia, high rates of MDR-cKp stool carriage [118] combined with high rates of HvKp stool carriage [66] and inadequate surveillance systems, create the perfect conditions for MDR-HvKp to develop and spread.

MDR Enterobacterales (including CRKp) are already a WHO critical priority pathogen for research and development. Southeast Asia is particularly vulnerable to the devastating effects of antimicrobial resistance [119]. Amongst MDR Enterobacterales, CR-HvKp and Hv-CRKp appear to be particularly worrying subtypes that have the potential for devastating infections in both community and hospital settings.

The shifting epidemiology of HvKp from being a community-acquired infection in healthy adults to a hospital-acquired infection in patients with co-morbidities has made the traditional clinical delineation of HvKp from cKp more difficult. Clinicians require either a clinical suspicion (which is now more blurred), or a timely identification of HvKp, for early and intensive investigation for metastatic spread, as well as to guide effective surgical treatment for the HMV phenotype and to guide the duration of antibiotic courses. This is increasingly difficult to achieve.

Even once a diagnosis of HvKp is made, treatment lacks a strong evidence base, with mortality >30% in a K1/K2 predominant *K. pneumoniae* case series [120] and up to 100% in MDR-HvKp [103] strains.

These challenges, already difficult for high-income countries (HICs) to address, will be amplified in LMICs, where resource availability may limit future genomic diagnostics, surveillance, access to antibiotics of last resort and timely surgical treatment.

## 4. Strategy

Both melioidosis and HvKp are neglected Gram-negative infections that have many parallels. In both diseases, patient outcomes suffer from a lack of awareness and evidence. Progress is hampered by a dearth of information on basic epidemiology, with a lack of regional or global surveillance systems. Furthermore, obtaining an accurate and timely diagnosis is challenging and there is a paucity of evidence for effective treatment. It is likely with improved diagnostics at regional and district levels, plus increasing awareness of these diseases amongst clinicians, that more cases are being detected, thus contributing to their rapid emergence. With further development and rollout of point-of-care testing, molecular testing, and/or mass spectrometry, the ability of local laboratories to diagnose these infections becomes easier, therefore contributing to the overall surveillance efforts of these diseases.

A significant milestone for these diseases would be their inclusion in the NTD list by the WHO. NTDs are a diverse group of conditions that are mainly seen in the tropics and affect poorer members of society with devastating health consequences (Table 3). Once a disease has been classified as an NTD, it becomes more visible to health partners and investors across many sectors, including medication development, and water and sanitation projects and is prioritised for research and funding. Melioidosis has much higher mortality and morbidity than many of the current NTDs, and it meets many of the criteria for being an NTD. It disproportionally affects those from a lower socioeconomic class, it is found in tropical and subtropical regions; strategies already exist for improved treatment and control, and it receives far less funding than other diseases with a similar health impact [121]. HvKp has a high mortality and morbidity, currently affecting predominantly tropical and subtropical regions; worryingly, HvKp does not appear to be bound by the tropical environment or population and is spreading globally. Whilst also affecting HICs, HvKp is present in LMICs, potentially to a greater degree [73,122]. The detection and management of HvKp is resource-intensive and the continued spread, particularly of MDR-HvKp, will disproportionately impact LMICs [123]. There is neither global data on the prevalence of HvKp, nor evidence for its optimum treatment. A lack of global consensus definition and research has hampered the proper understanding of its epidemiology, but effective (albeit evidence-scarce) treatment does exist, and evidence for control is largely extrapolated from the wealth of evidence for the control of other Enterobacterales.

A pre-requisite for WHO considering a disease as an NTD is the requirement for countries to have a robust routine surveillance system to capture the burden of disease. Currently, *B. pseudomdallei* is only notifiable in a few countries, such as Australia and Thailand. There are voluntary reporting networks such as “The International Melioidosis Network”, a Thailand-based, online registry that hopes to improve the understanding of the distribution and impact of disease [125]. Although these are useful data, they rely on individual centres voluntarily reporting cases and only record culture-confirmed cases, which is not always possible in endemic regions, leading to incomplete data. Requiring *B. pseudomallei* to be notifiable in high-prevalence countries would allow for a better understanding of the epidemiology and the identification of public health intervention targets and provide evidence to WHO to consider *B. pseudomallei* as an NTD.

At present, for HvKp, there are no formal surveillance systems in place, with only a few studies demonstrating passive surveillance [114,126]. Kleborate-viz [122] is a tool developed by Monash University that compiles genomic data for HvKp; however, this is purely voluntary. Therefore, there is a need for WHO-approved surveillance systems for both diseases.

**Figure 1:**
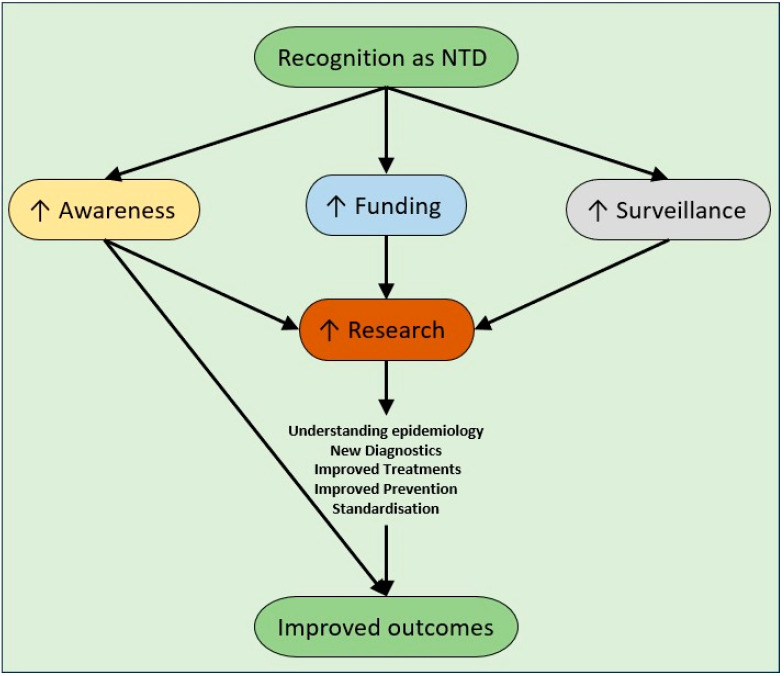
Expected benefits of recognition of melioidosis and hypervirulent *Klebsiella pneumoniae* as neglected tropical diseases.

There are many benefits in both diseases being recognised as NTDs (Figure 1). Firstly, awareness of both diseases would increase significantly, not just in endemic countries, but globally. Awareness needs to increase amongst clinicians, laboratory staff (especially for melioidosis to prevent occupational exposures), public health clinicians and governmental health agencies. Awareness would then lead to strengthened surveillance via clinical and laboratory reporting. Linked to this development are improving diagnostics, especially for resource-limited laboratories, where molecular diagnostics are preserved for national/regional reference laboratories due to resourcing. Rapid diagnostic tests will likely become a mainstay of diagnosis for areas where laboratory support is impractical.

Increased funding could also expedite further clinical trials for diagnosis and treatment. Further research is needed to establish the best treatment practices, including antibiotic combinations, treatment length, adjuncts such as G-CSF for melioidosis, and vaccination development. Ideally, these trials should be multicentre and international to allow for the development of global guidelines, following the success of the Recovery trial for COVID-19 [127].

## 5. Conclusions

Both melioidosis and HvKp pose significant health threats in Southeast Asia, and with increasing co-morbidities, particularly diabetes, it is anticipated that the prevalence of these diseases will significantly increase. Anti-microbial resistance will negatively impact the morbidity, mortality, and associated cost of treating these infections. Both diseases are unrecognised as NTDs, and as such, funding in relation to their surveillance and clinical research is minimal. Key priorities for these diseases include the development of WHO surveillance systems, the development of diagnostics, and treatment trials.

## Figures and Tables

**Table 1:** Antibiotic Treatment Guidelines. Adapted from the 2015 Darwin Guidelines [35].

Phase		Antibiotic	Adult Dose
Intensive		Meropenem	1 g IV TDS (2 g TDS if neurological involvement)
		Ceftazidime	2 g IV QDS
	Eradication	Co-trimoxazole	320 + 1600 mg PO BD (over 60 kg)
		Amoxicillin-clavulanate	20/mg/kg PO TDS
		Doxycycline	100 mg PO BD

IV: intravenous, TDS: three times daily, PO: orally, BD: twice daily.

**Table 2:** Recommended duration of antibiotic by site of infection; adapted from 2020 updated Darwin Guidelines [34].

Site of Infection	Minimum Intensive Phase Duration (Weeks)	Eradication Phase Duration (Months)
Cutaneous only	2	3
Bacteraemia without focus	2	3
Pneumonia without lymphadenopathy or ICU admission	2	3
Pneumonia with lymphadenopathy or ICU admission	4	3
Deep-seated collection	4 (from the last drainage sample growing *B. pseudomallei*)	3
Septic arthritis	4 (from the last drainage sample growing *B. pseudomallei*)	3
Osteomyelitis	6	6
Central Nervous System infection	8	6
Arterial infection or mycotic aneurysm	8	6

ICU: intensive care unit.

**Table 3:** Common Features of Neglected Tropical Diseases.

A proxy for poverty and disadvantage; Affect populations with low visibility and little political voice; Do not travel widely; Cause stigma and discrimination, especially of girls and women; Have an important impact on morbidity and mortality; Are relatively neglected by research; Can be controlled, prevented, and possibly eliminated using effective and feasible solutions.

From the first WHO report on neglected tropical diseases: working to overcome the global impact of neglected tropical diseases [124].
